# Behavioral and electrodermal data on implicit nocebo conditioning using supraliminally presented visual stimuli

**DOI:** 10.1016/j.dib.2019.104705

**Published:** 2019-10-22

**Authors:** Anne-Kathrin Bräscher, Michael Witthöft

**Affiliations:** Department for Clinical Psychology, Psychotherapy, and Experimental Psychopathology, Johannes Gutenberg University Mainz, Germany

**Keywords:** Nocebo effect, Implicit conditioning, Electrodermal activity, Contingency awareness, Pain perception

## Abstract

This article contains intensity and aversiveness ratings of electrical stimuli and data on electrodermal activity (skin conductance level and skin conductance response) during an implicit conditioning procedure. Further, answers from a questionnaire on contingency awareness are provided. The experiment consisted of three phases. In the acquisition, two types of visual stimuli (CS+ and CS-) were coupled to weakly and moderately painful electrical stimuli presented to the participants’ (*N* = 48) dominant hand. In the test phase, after both CS+ and CS- only the weakly painful electrical stimuli were presented. In the contingency test phase, no more electrical stimuli were presented and participants had the task to rate intensity and aversiveness as if an electrical stimulus had been presented. This phase served as a test for first-order contingency awareness. Afterwards participants filled in a questionnaire with five questions to assess their level of second-order contingency awareness. For more insight, please see Nocebo hyperalgesia induced by implicit conditioning (Bräscher and Witthöft, 2019).

**Table:** Specifications Table

Subject area	*psychology*
More specific subject area	*clinical psychology*
Type of data	*.csv-files (data files), .sps-files (SPSS syntax files), .doc-file (questionnaire)*
How data was acquired	*repeated visual analog scale ratings were acquired using Matlab (MATLAB and Data Acquisition Toolbox Release 2015b, The MathWorks, Inc., Natick, Massachusetts, United States); EDA was continuously recorded on the non-dominant hand with two Ag/AgCl electrodes (*24 mm*) and a sampling rate of* 32 Hz *(Varioport System, Becker Meditec, Karlsruhe, Germany*), questionnaire.
Data format	*raw, analyzed, preprocessed*
Experimental factors	*within-subject factors: cue (CS*+*, CS-), experimental phase (acquisition, test, contingency test), and trial (depending on the phase 1–10 or 1–15); between subjects covariate: contingency awareness*
Experimental features	*The main outcome measures were intensity and aversiveness ratings during an implicit conditioning design, while continuous EDA was recorded.*
Data source location	*Mainz, Germany*
Data accessibility	*data is with this article*
Related research article	Bräscher, A.-K. & Witthöft, M. nocebo hyperalgesia induced by implicit conditioning. *Journal of Behavior Therapy and Experimental Psychiatry 64*, 106–112. https://doi.org/10.1016/j.jbtep.2019.03.006

**Table:** 

**Value of the Data** - The data is useful as it allows exploration of implicit nocebo conditioning with supraliminally presented cues and electrical painful stimuli. - The data may be used to assess implicit conditioning effects in subjective ratings of intensity and aversiveness as well as electrodermal activity. - The data can be used for re-analysis, replication as well as meta-analytic analyses in the context of (implicit) conditioning and nocebo effects in pain perception.

## 1 Data

The data consists of a syntax file and.sav files with raw and averaged visual analog scale ratings on intensity and aversiveness and preprocessed and analyzed skin conductance level and skin conductance response recordings from a conditioning procedure and answer to a questionnaire assessing contingency awareness.

## 2 Experimental design, materials, and methods

### 2.1 Sample

The sample consisted of 48 healthy participants (M = 25.79 years, SD = 4.45; 25 females). Exclusion criteria comprised chronic or current acute pain, intake of pain medication or psychotropics, diabetes, hypertension, cardiopathy, thyroid disease, renal insufficiency, hepatic dysfunction, epilepsy, stroke, Parkinson's disease, multiple sclerosis, psychiatric or neurologic diagnoses, intake of illegal drugs, alcohol, medication, or drug abuse, pregnancy, and left-handedness. Participants gave written informed consent prior to the study. The experimental protocol was conducted in accordance with the Declaration of Helsinki (2008) and approved by the Local Ethics Committee.

### 2.2 Visual and electric stimuli

Weakly (low pain) and moderately painful electric stimuli (high pain) were applied to the dorsal index finger of the dominant hand and served as unconditioned stimuli (US).

Abstract pictures (black figures on white ground with red lines in the foreground) served as conditioned stimuli [1]. Pictures with a symmetrical black figure were coupled to high pain (CS+), whereas pictures with asymmetrical black figures were coupled to low pain (CS-). This assignment was counterbalanced across participants, i.e., for half of the participants, symmetrical figures serves as CS+ and for the other half asymmetrical figures served as CS+. Red lines in the foreground of the pictures served as distractors. There was no other differentiating feature (e.g. shape, complexity, percentage of white or black area, etc.) with regard to the contingency besides symmetry. A new picture was presented in every trial (in total 110 different pictures) in order to hinder the development of contingency awareness. Pictures were presented for 4 seconds and at a randomized time point within the last 2 s, the electric shock was applied (delay conditioning, i.e., the US is presented during the presentation of the CS).

### 2.3 Stimulation device

Electrical stimuli were delivered to the dorsal index finger of the dominant hand, through a pair of Ag/AgCl electrodes by a bipolar constant-current stimulator (DS5; Digitimer, Welwyn Garden City, Hertfordshire, UK). The stimulator was coupled to a data acquisition system (DT9812-10V; Data Translation, Inc., Marlborough, Massachusetts, United States), which was controlled by a laptop computer. Each stimulus consisted of an individually calibrated sinus wave pulse with a duration of 500 milliseconds, defined in MATLAB (MATLAB and Data Acquisition Toolbox Release 2015b, The MathWorks, Inc., Natick, Massachusetts, United States).

### 2.4 Calibration procedure

The electric stimuli (low and high pain) were calibrated to the participants’ individual intensity level of weak and moderate pain. A sequence of electric stimuli was applied, starting with 0.25 mA and increasing in 0.25 mA steps in every trial. Each stimulus was rated on the intensity VAS and the sequence was stopped as soon as VAS score greater than 75 was reached. This procedure was repeated three times. The level of the low pain stimulus was identified by calculating the mean mA of those electric stimuli that were evaluated between 40 and 50 on the VAS. The level of the high pain stimulus was identified by calculating the mean mA of those electric stimuli that were evaluated between 65 and 75 on the VAS. The average intensities of the applied electric shocks were 1.76 mA (*SD* = 1.03) for the low pain stimulus and 2.24 mA (*SD* = 1.18) for the high pain stimulus.

### 2.5 Psychophysical scales

Participants were then familiarized with two horizontally oriented visual analog scales (VAS) in order to independently rate intensity and aversiveness of the electric stimuli. The intensity scale was labelled with 0 ‘not detectable’ and 100 ‘very painful’. At a scale value of 40, an additional anchor was included, labelled ‘just painful’ [2]. The aversiveness scale had the descriptors 0, ‘neutral’ and 100 ‘very aversive’.

### 2.6 Experimental design and procedure

Participants took part in one experimental session of approximately 45 min duration and were told that the purpose of the experiment was to investigate the impact of visual stimuli on pain perception. They completed calibration, the conditioning procedure, and the contingency test phase. Afterwards, they answered the post-experimental questions in order to assess second-order contingency awareness (i.e., being aware of being aware).

The conditioning procedure started with the acquisition phase, in which the high pain stimulus was applied during the presentation of the CS+ and the low pain stimulus was applied during the presentation of the CS- in 60 trials. In the subsequent test phase, only low pain stimuli were applied both during the presentation of CS+ and CS- in 30 trials. After that, the contingency test phase followed with 20 trials, in which no electric stimulus was applied during and the participants were asked to indicate their sensation as if a stimulus had been presented. This sequence served to test first-order contingency awareness (i.e, awareness that does not need introspection, cf [3]. The sequence of CS- and CS + trials was randomized within each phase.

Every trial of the acquisition and test phase started with the presentation of a CS (4 seconds), during which an electric stimulus was applied (the exact time point of the electric shock was randomized within the last 2 seconds of the presentation of the CS). Then, participants rated their subjective sensation of the electric stimulus on the intensity and the aversiveness VAS. A fixation cross (4–6 seconds) followed before the start of the next trial. In the contingency test phase, no electric shock was applied and the fixation cross was only shown for 2 seconds, nothing else was changed.

### 2.7 Post-experimental questionnaire

After the experiment, participants answered a series of questions in a funnel debriefing manner (i.e., asking increasingly specific questions; [4]) in order to assess second-order awareness [3]. The post-experimental questionnaire consisted of seven questions. First, participants were asked about their thoughts on the research question (open question). Most participants assumed that the focus of the experiment was to investigate the influence of picture viewing on pain perception (*n* = 33, 70%). Seven out of 48 participants (15%) assumed some kind of conditioning experiment without being able to specify this, four participants (9%) had no idea and two (4%) had other explanations. Then they were asked whether the respective picture shown in a trial somehow was related to the intensity of the following electric shock (yes/no) and if so how exactly (open question). Then they were asked to indicate their agreement or disagreement (yes/no) with the following sentences: “I think that stronger electric shocks usually came after a picture with a symmetrical black figure in the background”, “I think that stronger electric shocks usually came after a picture with an asymmetrical black figure in the background”, “I think that weaker electric shocks usually came after a picture with a symmetrical black figure in the background”, and “I think that weaker electric shocks usually came after a picture with an asymmetrical black figure in the background”.

### 2.8 Electrodermal activity (EDA)

EDA was recorded continuously on the non-dominant hand with two Ag/AgCl electrodes (24 mm) and a sampling rate of 32 Hz (Varioport System, Becker Meditec, Karlsruhe, Germany). The data was baseline and range-corrected and further analyzed using the Matlab-based software Ledalab 3.4.9 ([5]; www.Ledalab.de). Preprocessing involved downsampling to 16 Hz and filtering with a unidirectional 1st order Butterworth low pass filter with a cut off frequency of 5 Hz. The data was visually checked for artefacts. EDA was analyzed by means of continuous decomposition analysis (CDA) with a response window of 1–4 seconds after the onset of the respective picture and an amplitude threshold of 0.01 μS. This time interval corresponds to the first interval response (FIR), which in differential conditioning designs has shown to be most effective in detecting conditioned responses. Ledalab returns various parameters of phasic and tonic activity, of which CDA.SCR (phasic activity within the response window; skin conductance response) and CDA.Tonic (decomposed tonic component within the response window; skin conductance level) were further analyzed.

### 2.9 Statistical analyses

Due to equipment failure, one participant's answers to the post-experimental questionnaire were not saved properly, resulting in missing data.

For identification of participants who showed first-order contingency awareness in the contingency test phase, the reliable change index (RCI; [6]), was calculated using Cronbach's α of the respective ratings of the low stimulus during the acquisition phase to determine reliability (*r*_tt_; [7]). Coming from psychotherapy research, the RCI measures whether a change in a person's score from one assessment to the next is statistically significant, i.e., larger than expected by chance, considering the reliability of the measuring instrument. As values > 1.96 indicate a significant change of the individual, participants with an RCI >1.96 were excluded in those subsequent analyses that tested for the conditioned effect in first-order contingency unaware participants.

Summing the correct answers to the five yes/no questions in the post-experimental questionnaire (Cronbach's α = 0.64), an index of second-order awareness was calculated (awareness index), assuming that a larger index corresponds to a higher degree in second-order contingency awareness.

Repeated measures ANOVAs for the subjective ratings and skin conductance level with the factors ‘cue’ (CS+, CS-) and ‘experimental phase’ (acquisition, test phase, contingency test phase) were used to assess differential responding to trials cued with CS+ and CS-. Excluding participants who became contingency aware according to the contingency test phase, repeated measures ANOVAs followed for the test phase, including the factors ‘cue’ and ‘trial’ (one to fifteen) in order to test for possible extinction. Then, the centered awareness index was added as a covariate (for the whole sample) and the conditioned effect was tested again assessing whether second-order contingency awareness is a necessary condition for the conditioned nocebo effect.

Finally, skin conductance levels (SCL) of CS+ and CS- trials, respectively were predicted with multiple linear regressions using both intensity and aversiveness ratings as predictors in order to confirm the validity of the results of previous analyses showing significant results for aversiveness but not intensity ratings.
