# Supplementary material for: Behavioral and electrodermal data on implicit nocebo conditioning using supraliminally presented visual stimuli
Source: Data Brief. 2019 Oct 22;27:104705. doi: 10.1016/j.dib.2019.104705 (PMC6838380; doi:10.1016/j.dib.2019.104705)
Supplement: Multimedia component 1 [file mmc1.docx]

**Post-experimental questionnaire**

1. What, do you think, was the purpose of the experiment? (free text)
2. Was the respective picture shown in a trial somehow related to the intensity of the following electric shock? (yes/no)

If yes, please explain how the picture was related to the following electric shock. (free text)

1. I think that stronger electric shocks usually came after a picture with a symmetrical black figure in the background. (yes/no)
2. I think that stronger electric shocks usually came after a picture with an asymmetrical black figure in the background. (yes/no)
3. I think that weaker electric shocks usually came after a picture with a symmetrical black figure in the background. (yes/no)
4. I think that weaker electric shocks usually came after a picture with an asymmetrical black figure in the background. (yes/no)
